# Sensing in Urinary Catheter Systems: From Measurands and Signal Fidelity to Supportable Clinical Claims

**DOI:** 10.3390/s26175659

**Published:** 2026-09-06

**Authors:** Shuai Zhang

**Affiliations:** School of Pharmacy, Queen’s University Belfast, Belfast BT9 7BL, UK; shuai.zhang@qub.ac.uk

**Keywords:** urinary catheter, sensor, crystalline encrustation, blockage warning, catheter-associated urinary tract infection, biofilm, sensor-integrated biomaterials, multimodal sensing, reference endpoint, clinical translation

## Abstract

This review evaluates urinary catheter sensors designed to warn of crystalline encrustation and blockage or to monitor infection-related microbial, interfacial, and host-response states. A source-to-claim framework links each measured state and sampling compartment with its readout, reference endpoint, and supportable clinical claim. The analysis also considers whether catheter integration preserves the relationship between source, readout, and claim. pH-responsive systems have the strongest human feasibility evidence, although the studies are small, use different sampling configurations, and include few blockage events. Evidence for urease sensing is confined to analytical studies and controlled in vitro experiments. Mineral-deposition and hydraulic approaches rely mainly on catheter models or adjacent-device studies. Most infection-related platforms are sample-based assays, prototypes, or short-duration systems that measure clinically non-equivalent states. Analyte detection alone cannot establish a diagnosis or predict a later event in either application. Interpretation also changes with sensor location and with the effects of transport, fouling, and mechanical loading on the recognition interface. Translation requires validation over complete catheter episodes against an endpoint matched to the intended claim. An additional channel is warranted only if it resolves a prespecified uncertainty and improves clinically relevant performance against a fixed comparator.

## 1. Introduction

Indwelling urinary catheters are widely used in acute and long-term care [1,2], but their use is frequently complicated by catheter-associated urinary tract infection (CAUTI), crystalline encrustation, and impaired drainage [1,2,3]. Historical community studies reported recurrent obstruction in approximately 40–50% of long-term catheter users [3], although current prevalence across care settings is uncertain. These complications can cause pain or urine leakage and may lead to emergency catheter replacement or unnecessary antimicrobial exposure [1,2,3]. Clinically, recurrent blockage is usually managed only after symptoms or impaired drainage appear [3,4]. By contrast, CAUTI follows a different clinical pathway. The National Institute for Health and Care Excellence (NICE) defines CAUTI as a symptomatic infection of the bladder or kidneys in a person with a urinary catheter and notes that nearly all users have bacteriuria after 1 month [5]. Antibiotic treatment is not routinely indicated in the absence of symptoms. When CAUTI is suspected, a fresh urine sample should be collected before antibiotics and sent for culture and susceptibility testing [5]. Earlier blockage sensing could allow planned assessment or catheter exchange before drainage fails completely. An infection-related alert could instead prompt symptom-led evaluation and microbiological sampling before treatment, consistent with infection-prevention and antimicrobial-stewardship priorities [6,7]. However, whether either approach changes clinical decisions or patient outcomes remains unknown.

Once inserted, the catheter becomes a dynamic host–material–microbial interface. Host-derived molecules and microbial products adsorb onto the surface and form a conditioning film that alters subsequent bacterial adhesion, mineral nucleation, and electrode behavior [8,9]. Catheterization also changes the bladder environment and host response [10,11]. Geometry and flow vary along the drainage pathway, producing local differences in transport, residence time, and wetting [1,12,13]. Sensor position therefore determines the sampled compartment and the extent to which transport and aging separate the measured signal from the event of interest.

The clinical meaning of a sensor result depends on the stage of infection or device failure being measured. Infection-related sensing covers clinically non-equivalent states such as organism detection, bacteriuria, catheter-surface biofilm, host inflammation, and symptomatic CAUTI [5,14,15]. Urease-driven crystalline encrustation, by contrast, proceeds through a more ordered biochemical-to-hydraulic sequence [16,17,18,19,20,21,22,23]. Biofilm and mineral deposits develop together [24,25], and drainage can also fail through noncrystalline mechanisms [4]. The validation endpoint should therefore match the measured state rather than a generic claim of early detection.

Recent catheter-integrated materials and connected drainage technologies extend urinary sensing beyond visual indicators [26,27,28,29]. Existing reviews mainly classify these technologies by device type or sensing modality [1,28,30,31]. This review instead examines how the measured state, sampling compartment, material architecture, and integrated-device readout constrain the clinical claim that the evidence can support. It also asks whether an additional channel contributes information beyond a fixed comparator over a complete catheter episode. Blockage prediction is within scope as an intended claim, but observed threshold-to-blockage intervals are distinguished from validated episode-level prediction. Prediction of symptomatic CAUTI is outside scope because it would require a prespecified horizon and an independently adjudicated incident endpoint.

The source-to-claim chain is a catheter-specific synthesis rather than a new general validation theory. It brings together context-of-use terminology from BEST, diagnostic-accuracy and comparative-testing principles, device-development staging in IDEAL-D, and standards-based device requirements [32,33,34,35,36,37,38,39,40]. Its catheter-specific contribution is to make the sampling compartment and integration-dependent signal fidelity explicit. For each platform, the analysis identifies the measured state and sampling site, examines how catheter integration changes the readout, and determines which claim the reference endpoint supports. Signal fidelity refers to preservation of the relationship between the target measurand and final readout after integration. General physiological monitoring is excluded unless flow, pressure, or contact directly indicates drainage impairment or affects validity of the primary signal. Evidence from adjacent systems is used only to establish engineering feasibility.

### Review Approach and Evidence Selection

This critical narrative review was updated on 1 September 2026. PubMed/MEDLINE and Google Scholar were searched without a start-date restriction through 27 August 2026. Targeted checks of relevant standards and regulatory guidance were completed on 1 September 2026. Publisher and DOI searches and backward and forward citation tracking were used to capture engineering studies not consistently indexed in biomedical databases. The core search combined (“urinary catheter” OR “Foley catheter” OR “catheter bag” OR “urinary drainage”) with (sensor OR sensors OR biosensor OR biosensors OR monitoring OR detection OR warning OR “smart catheter”) and with (blockage OR encrustation OR urease OR pH OR biofilm OR infection OR CAUTI OR impedance OR biomarker OR flow OR pressure). Targeted searches were then run for named materials, analytes, devices, and trial identifiers.

English-language primary studies were included when they evaluated a urinary catheter, drainage pathway, catheter bag, or clearly relevant adjacent urinary device and reported a measurable sensor response, material architecture, or clinical endpoint. General urinalysis, non-urinary catheter sensing, and antimicrobial or antifouling devices without a sensing function were excluded, except when needed to explain signal validity or failure mechanisms, or to provide relevant off-device comparators. Authoritative guidelines, standards, and trial registries were included for definitions, device requirements, and unpublished study status. Gray literature was limited to a preprint reporting scarce human catheter-blockage data [41], registered trials [42,43], and applicable standards. Commercial claims and nontechnical webpages were excluded. Study selection and interpretation were performed by the single author. Because the purpose was critical synthesis rather than effect-size pooling, no meta-analysis or formal risk-of-bias score was applied. Evidence-gap statements are restricted to studies meeting these criteria and are phrased as findings of the included evidence rather than universal absence claims.

## 2. Encrustation and Blockage Sensing: From Urease Activity to Drainage Failure

In crystalline encrustation, urease-producing uropathogens hydrolyze urea to ammonia and raise urinary pH [16,17,21,22]. The resulting alkaline conditions promote struvite and calcium phosphate precipitation within the developing biofilm [18,19,20,23]. Mineral deposition varies in rate and location with urine chemistry, local transport, and biomass [4,18,19,20]. The portion of this heterogeneous process that is sampled also depends on sensor location. As biofilm and mineral deposits narrow the drainage eyelets and lumen, sensors can detect changes in chemistry, surface accumulation, and drainage.

**Figure 1:**
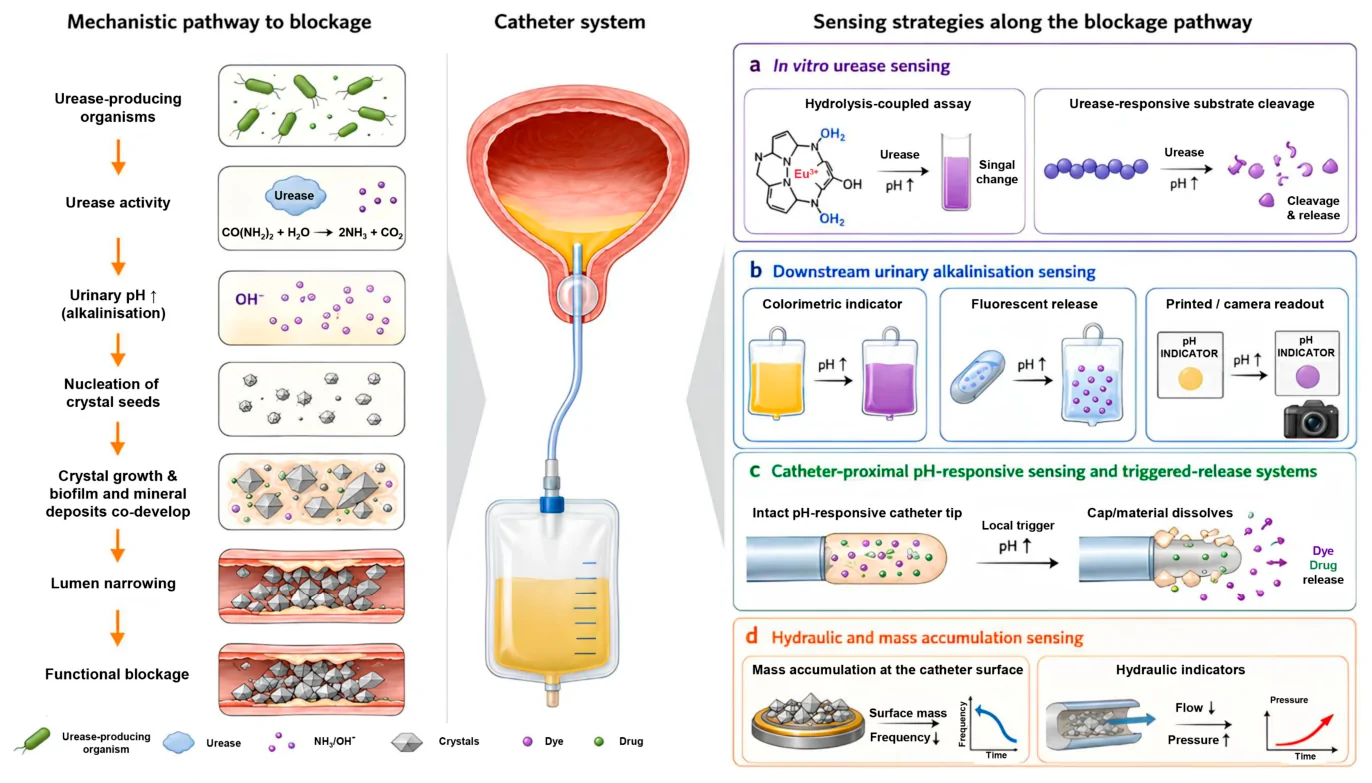
Mechanistic pathway and sensing positions for urinary catheter blockage. The left-hand sequence follows urease-producing organisms through urease activity, urinary alkalinization, crystal nucleation, co-development of biofilm and mineral deposits, lumen narrowing, and functional blockage. The central schematic locates the catheter within the bladder-to-drainage-bag system. (**a**) In vitro urease sensing by a hydrolysis-coupled assay or urease-responsive substrate cleavage. (**b**) Downstream urinary alkalinization sensing by a colorimetric indicator, fluorescent release, or printed/camera readout. (**c**) Catheter-proximal pH-responsive sensing and triggered release of dye or drug. (**d**) Catheter-surface mass accumulation and hydraulic impairment monitored through changes in resonance frequency, flow, or pressure. The schematics indicate sensing position and transduction concept rather than comparative performance.

Current designs target functional urease activity, urinary alkalinization, mineral deposition, or late hydraulic impairment. At the alkalinization stage, downstream devices report bulk urinary pH, whereas catheter-proximal systems may signal the same change or trigger release. The sequence used here follows mechanistic position, not reported performance. Upstream signals may allow more time for assessment, but downstream measurements lie closer to functional impairment. A claimed gain in threshold-to-blockage interval is meaningful only when measured from a prespecified sensor threshold to a defined blockage or flow-cessation endpoint. Mass transport, biofouling, and mechanical loading can alter signal generation and interpretation at any stage. Figure 1 maps these sensing positions onto the mechanistic pathway, from upstream urease activity to surface-mass and hydraulic changes near functional impairment.

Three timescales need to be distinguished. Material response time is the interval between a defined stimulus and a detectable output under specified conditions. Threshold-to-blockage interval runs from a sensor reaching its prespecified threshold to the study-defined blockage or flow-cessation endpoint. The prediction horizon is the prespecified period after an alert within which a later blockage event is counted. Rapid material response does not necessarily provide useful advance warning. Episode-level predictive value also depends on how consistently blockage occurs within the chosen horizon.

### 2.1. Functional Urease Sensing

Functional urease sensors target an upstream catalytic event using two main formats (Figure 1a). Hydrolysis-coupled assays infer enzyme activity from the chemical or physical products of urea breakdown. Enzyme-responsive substrates use urease-catalyzed cleavage or degradation to release or dequench a reporter. Both formats measure functional urease activity rather than organism identity. Buffering, matrix transport, and substrate degradation affect their outputs in different ways.

Evidence comes from analytical studies and controlled in vitro proof-of-concept experiments [44,45,46]. Surender et al. reported a supramolecular europium(III)-cyclen complex sensor that showed urease-dependent luminescence quenching in vitro and remained responsive after incorporation into a hydrogel [44]. A modified Christensen-broth assay characterized species- and concentration-dependent urease kinetics among common catheter-associated uropathogens but did not assess catheter-integrated sensing [46]. In another design, urease cleaved urea-containing units within fluorescein-labeled polymeric particles, separating initially self-quenched fluorophores and increasing fluorescence. Urease-positive *Klebsiella pneumoniae* and *Enterobacter cloacae* were detected within 5 h under controlled conditions [45]. The 5 h interval describes material response, not warning before blockage. Optical thresholds from these formats may represent different urease activities or microbial burdens.

Once integrated into a catheter, the catalytic response reflects mass transport as well as enzyme activity. Matrix permeability, hydration, and thickness influence substrate access and reporter generation. Urinary buffering can also decouple enzyme activity from the resulting pH change. Because kinetics vary by species and concentration [46], the same reporter threshold may represent different biological burdens. These studies establish that functional urease activity can be measured early in the encrustation pathway. Whether this produces useful warning is unknown because no head-to-head catheter study has compared threshold-to-blockage intervals or episode-level prediction. Clinical value would require the signal to distinguish episodes that progress to mineralization or loss of patency from those that do not.

### 2.2. Downstream pH-Responsive Sensing

pH-responsive systems detect urinary alkalinization downstream of microbial urease activity and have the most extensive human feasibility evidence among the strategies reviewed (Figure 1b). Available formats include immobilized colorimetric indicators read visually or by absorbance [47,48], release-based designs in which concentrated carboxyfluorescein is retained behind an alkaline-responsive barrier [49,50], and printed labels coupled with camera-based analysis [51]. These devices can be placed at the catheter–drainage-tube junction, along the drainage line, or in the collection bag. At each location, they report bulk urinary alkalinization in the sampled drainage compartment and remain accessible for external readout.

Indicator pKa and optical contrast set the nominal sensing range, but immobilization can shift the apparent transition through ion partitioning. Matrix charge and hydration also contribute to this shift. Physical entrapment risks indicator leaching. Covalent immobilization improves retention but can restrict analyte access. These effects should be characterized for each material architecture [47,48,51]. Retained indicators respond through wetting, ion diffusion, and optical equilibration. Release-based systems also depend on barrier activation and downstream reporter transport and are generally single-use. The operating threshold and observed threshold-to-blockage interval reflect the full sensing architecture rather than indicator chemistry alone.

Across continuous-flow catheter models, reported threshold-to-blockage intervals ranged from several hours to approximately 43 h [47,48,49,50,51]. Direct comparison is not possible because device configuration and endpoint definition differed. Bag-mounted sensors measure urine after downstream transport and storage [12,49,50,51]. Junction sensors sample fresher urine but may be wetted intermittently [47]. In one model, the bag sensor responded earlier because the junction device was not continuously wetted [47]. The observed warning interval therefore reflects the material-fluidic configuration more than simple distance from the catheter.

Small cohorts and few blockage events preclude precise estimates of catheter-episode predictive performance. In the 2006 study, 20 participants were followed through one catheter lifetime, so each participant contributed one catheter episode. All 15 participants colonized with *Proteus mirabilis* produced a positive response, and 12 later experienced blockage. The mean signal-to-blockage interval was 12 days [52]. The 2014 study recruited 17 participants and followed 11 prospectively to catheter change. Three episodes ended in blockage on days 22, 23, and 25, and the study reported sensor color change within 24–48 h after insertion [53]. URINOSTICS used samples rather than catheter episodes as the unit of analysis. It evaluated 35 donated catheter and drainage-bag samples from 28 individuals, including repeat donations, against a prespecified three-week prediction horizon [41,42]. Both observed blockage events were detected, and specificity was 58.1% at the donation level. With repeat donations and only two blockage events, this estimate cannot be compared directly with catheter-episode predictive performance.

The human studies demonstrate feasibility, although their outcomes are not directly comparable. The 2006 and 2014 studies related sensor signaling to later catheter outcomes in followed participants, whereas URINOSTICS classified donated samples against a three-week prediction horizon. None estimated a calibrated probability of blockage or a reliable time to event across independent catheter episodes. These data support pH-responsive devices as alkalinization alerts that can prompt review or confirmatory assessment. Because alkalinization is upstream and potentially reversible, the signal identifies conditions favorable for crystallization without showing whether or when mineral deposition will cause clinically significant encrustation or drainage failure.

### 2.3. Catheter-Proximal pH Sensing and Theranostic Triggered-Release Systems

At the intravesical catheter interface, the same alkaline shift can be measured locally (Figure 1c). A signal-only system reports the change without altering subsequent blockage progression. Milo et al. developed a coating in which self-quenched 5(6)-carboxyfluorescein was encapsulated in a poly(vinyl alcohol) base layer beneath a pH-responsive Eudragit S100 cap. When urinary pH exceeded 7.0, the cap dissolved and released dye into the drainage stream. Under continuous flow, the visible signal preceded *Proteus mirabilis*-mediated blockage by up to 12 h, and the coating remained intact under sterile conditions and with urease-negative bacteria [26]. Because the experiment did not reproduce prolonged clinical catheterization, long-term stability and warning performance still require evaluation in humans.

In a release-based warning system, response time includes barrier activation and reporter transport. Barrier thickness, permeability, and swelling govern the balance between activation speed and reporter retention. A thin or highly permeable barrier can accelerate release at the cost of passive leakage and premature depletion. A denser barrier improves retention but may delay or limit reporter release. These systems demonstrate pH-triggered activation under the tested conditions, although the contribution of each reaction and transport step to the overall response time remains unresolved.

When the pH trigger also releases a treatment, time to blockage reflects both sensing and treatment effects. A multilayer chitosan/poly(acrylic acid) coating containing ciprofloxacin-loaded polydiacetylene vesicles changed color, accelerated drug release, and maintained lumen patency for more than 30 h in vitro [27]. A dual-action platform combined carboxyfluorescein with either acetohydroxamic acid or ciprofloxacin. In an in vitro *Proteus mirabilis* model, the ciprofloxacin coating produced a mean threshold-to-blockage interval of approximately 79 h and extended functional catheter lifetime 3.4-fold [29]. A poly(vinyl alcohol)/Eudragit reservoir released *Proteus mirabilis* bacteriophages after alkalinization, reducing bacterial burden and extending reported time to blockage from 13 to 26 h [54]. That reservoir had no independent warning readout. Because treatment changes blockage kinetics, the longer patency in these systems cannot be attributed to earlier sensing alone. Their clinical intervention window has not been evaluated in humans.

### 2.4. Mineral-Deposition and Hydraulic Sensing

Mineral-deposition sensors operate farther downstream, after urinary alkalinization but before complete occlusion, as represented by the surface-mass branch of Figure 1d. In an adjacent ureteral-stent model, urease was applied to a polymer-coated quartz crystal microbalance (QCM) to induce calcium- and magnesium-phosphate deposition from urine [55]. The use of a ureteral stent means that the result establishes engineering feasibility but not validity for urinary catheter blockage. Direct validation will require testing in a representative Foley-catheter system. QCM infers total surface-coupled mass from a resonant-frequency shift, so the response is not mineral-specific. Conditioning-film components and biofilm can also add mass. Differences in surface chemistry and roughness between the sensing element and catheter further affect nucleation and adhesion. A mineral-specific claim requires independent compositional confirmation and testing on representative catheter surfaces.

The hydraulic branch of Figure 1d draws on studies in adjacent systems that have demonstrated continuous urine-output measurement [56,57,58,59], bladder or intra-abdominal pressure monitoring [60,61], and sensor-based drainage monitoring during continuous bladder irrigation [62]. These other indications establish engineering feasibility but do not validate the signals for encrustation-related blockage. Flow and pressure change near functional failure, yet similar changes can arise from altered urine production, bladder activity, drainage-system mechanics, or noncrystalline narrowing. Attribution to crystalline encrustation therefore requires non-encrustation controls and independent confirmation of mineral deposition. An integrated catheter must also meet ISO 20696:2018 requirements and test methods [63]. Compliance with that standard does not establish predictive validity for the sensing function.

Urease activity and pH are upstream biochemical states that may offer more time for assessment, although they have limited event specificity. Mineral deposition, flow, and pressure occur closer to functional failure but do not identify crystalline encrustation as the cause. In the included evidence, human studies evaluated pH-responsive strategies alone [41,52,53], whereas model-based combined platforms linked pH-triggered sensing to treatment or reporter release rather than testing incremental prediction [27,29]. No study compared a prespecified biochemical–physical combination with the same fixed upstream sensor across complete catheter episodes. Incremental episode-level value therefore remains unestimated. Table 1 compares sensing architectures, evaluated endpoints, and current claim boundaries.

## 3. Infection-Related Sensing: Bulk Signals, Microbial Targets, Interface State, and Host Response

**Figure 2:**
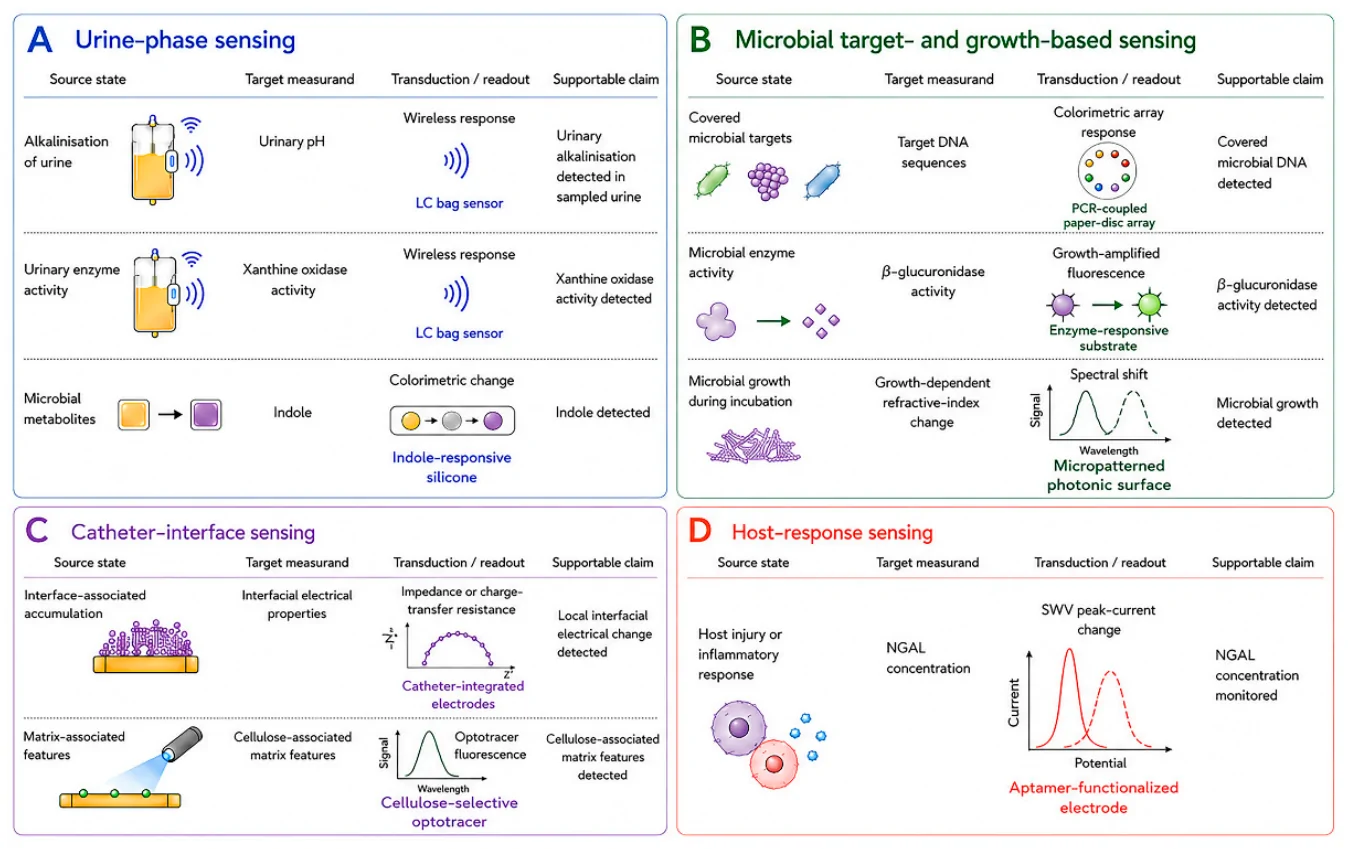
Infection-related sensing categories and evidence-supported claim boundaries in urinary catheter systems. (**A**) Urine-phase pH/xanthine oxidase LC bag sensors and indole-responsive silicone [64,65]. (**B**) Microbial target- and growth-based sensing by PCR-coupled paper-disc arrays, growth-amplified β-glucuronidase fluorescence, or growth-dependent photonic shifts [66,67,68]. (**C**) Catheter-interface sensing by impedance/charge-transfer resistance or cellulose-matrix optotracing [69,70,71,72,73,74]. (**D**) Host-response sensing of NGAL with an aptamer-functionalized catheter electrode and SWV readout [75]. Each panel distinguishes source state, measurand, readout, and evidence-supported claim. Illustrations and traces are schematic. LC, inductor-capacitor; NGAL, neutrophil gelatinase-associated lipocalin; PCR, polymerase chain reaction; SWV, square-wave voltammetry.

Infection-related sensing spans several biological states rather than a single progression from an early biochemical signal to a defined clinical event. The systems reviewed here measure urine chemistry or metabolites, selected microbial targets, catheter-associated accumulation, or host injury and inflammation. Detection at any of these levels does not by itself establish CAUTI diagnosis or prospective prediction. Figure 2 organizes these four source-state categories and the current claim boundary for each.

### 3.1. Urine-Phase Physicochemical and Metabolic Sensing

Urine-phase systems measure physicochemical conditions or diffusible microbial metabolites in flowing or collected urine, not changes on the catheter surface (Figure 2A). They can be placed at the catheter wall, in the tubing, or in the collection bag. Bag-based formats avoid an additional intravesical component and can support reusable external readers. Rana et al. integrated a passive wireless inductor-capacitor sensor into a drainage bag and detected experimentally elevated pH (>8) or xanthine oxidase (XOD) activity (>500 U/L) within 3 h [64]. The disposable tag used laser-patterned aluminum traces on flexible metallized polyethylene terephthalate (PET), with stimuli-responsive polymer coatings on interdigitated electrodes connected to planar coils. Concurrent measurement of two analytes is an appropriate early-stage engineering proof of concept. The Biomarkers, EndpointS, and other Tools (BEST) Resource is used here to define the evidentiary step needed for a higher-level claim, not as a prerequisite for reporting the prototype [32]. BEST defines a multicomponent biomarker as specified inputs combined by a defined rule or algorithm to produce an interpretable output. Accordingly, this device is described as a dual-analyte sensing platform. A claim of a multicomponent biomarker or CAUTI classifier would additionally require a prespecified combination rule, independent validation, and comparison with each single-channel baseline. The two analytes would also need to contribute distinct information to the intended decision.

Brady et al. impregnated S-nitroso-N-acetylpenicillamine (SNAP) into silicone and reported colorimetric responses to indole-spiked urine and *Escherichia coli* cultures [65]. Indole partitions into the silicone and reacts within the matrix to form indoxyl red. The sensor therefore identifies indole production under the test conditions rather than *Escherichia coli* specifically. In this architecture, silicone serves as the structural substrate, analyte-partitioning phase, and reaction medium rather than being covered by a separate selective topcoat. Thickness, reagent loading, oxygen permeability, deformation, reagent depletion, and surface contamination can all alter response. Compared with pH, indole narrows microbial coverage to producing organisms but still cannot distinguish catheter colonization, asymptomatic bacteriuria, and symptomatic CAUTI. The available results establish controlled detection of predefined urinary chemistry or metabolites. Long-dwell reagent retention and clinical triage performance were not evaluated.

### 3.2. Organism-Targeted, Molecular, and Growth-Based Microbial Sensing

Organism-targeted systems provide greater microbial attribution than bulk-urine chemistry by measuring selected microbial enzyme activities, nucleic-acid sequences, or growth under defined culture conditions (Figure 2B). Each readout answers a different question. Enzyme assays report catalytic activity, PCR detects a covered genomic target, and growth-based methods show whether organisms proliferate under the assay conditions. Xu et al. attached a flexible fluorescence reader to a catheter bag containing enzyme-responsive substrates [66]. Product formation during post-collection growth generated a β-glucuronidase-dependent signal, with higher starting concentrations producing earlier responses. The observed response time reflected post-collection growth and enzyme-dependent product accumulation rather than catheter-associated burden at sampling. Specificity was assessed against a limited range of non-target organisms, and some *Escherichia coli* isolates lack the relevant enzyme. Although the study estimated starting inoculum from time to detection under controlled growth, that relationship was not validated quantitatively in clinical catheter samples.

Polymerase chain reaction (PCR)-based assays address a different question by detecting selected microbial DNA targets. Li et al. combined PCR with a paper-disc colorimetric array to identify 12 prevalent UTI-associated bacterial pathogens in clinical urine samples [67]. Multiplexing broadens coverage beyond one enzyme target, but the assay remains limited to organisms represented in the primer panel and may miss uncommon pathogens. A positive result confirms the presence of a covered genomic target in extracted urinary DNA. Establishing organism viability or catheter-surface colonization requires additional evidence.

iPRISM, by contrast, captures microorganisms on an amine-functionalized, micropatterned silicon photonic surface and detects growth-dependent changes in reflected-light interference during incubation. At the prespecified 90 min threshold, the assay achieved 97% sensitivity and 60% specificity against standard clinical culture [68]. The later 75 min decision point, which yielded 82% sensitivity and 81% specificity, was selected and evaluated post hoc in the same dataset. It is therefore exploratory and susceptible to optimistic bias or overfitting. The prespecified 90 min result should be treated as primary until the shorter threshold is validated independently. Each urine specimen was analyzed as a separate sample rather than as part of a longitudinal catheter episode, and reference classification relied mainly on culture rather than independent clinical adjudication of symptomatic CAUTI. Unlike panel-based PCR, iPRISM is not restricted to predefined primer targets, although species identification requires a separate method. Neither platform has been evaluated in situ or longitudinally over a complete catheter episode.

Conventional dipsticks and whole-urine spectroscopic assays provide off-device comparators for nitrite, leukocyte esterase, or composite optical signatures [76,77,78]. Their reported performance comes from isolated samples and does not establish continuous or longitudinal monitoring in a catheter system. Catheter integration changes analyte handling and the measurement environment, so benchtop performance cannot be assumed to transfer. Molecular assays require reliable nucleic-acid extraction and inhibition control. Photonic and affinity formats are vulnerable to nonspecific deposition and fouling. Performance must therefore be tested at the intended sampling location under representative flow and dwell conditions. Even when analytically valid, these methods detect only a covered molecular target, selected enzyme activity, or growth response. They cannot separate transient bacterial presence, catheter colonization, asymptomatic bacteriuria, and symptomatic CAUTI. Their likely role is organism attribution followed by confirmatory assessment rather than standalone CAUTI diagnosis. Interface sensors address a different question by measuring change directly at the catheter surface.

### 3.3. Catheter-Interface State and Biofilm-Associated Sensing

Catheter-interface sensors measure electrical or optical changes associated with microbial attachment and material deposition (Figure 2C). Their response to surface-associated change may cover a wider range of organisms than a single microbial target, although extracellular matrix, adsorbed host material, and mineral deposits can also contribute. One platform used a 25.4 μm flexible polyimide film carrying chromium/gold interdigitated electrodes (20/200 nm metal thickness, 300 μm line width and spacing) inserted along the catheter lumen. Nonsensing traces were passivated with parylene-C while the active electrode area remained exposed [69,70,71]. The platform showed decreasing impedance during *Escherichia coli* biofilm formation [69]. Flexible polyimide allows conformity to the cylindrical lumen, but long-dwell adhesion to silicone, edge sealing, cyclic bending, and stability of the exposed gold area were not established. A later Foley-catheter prototype preserved drainage and balloon inflation while operating wirelessly in a short model-bladder study [70,71]. In an adjacent system, gold electrodes showed increasing charge-transfer resistance during *Staphylococcus epidermidis* biofilm formation [72]. Total impedance and charge-transfer resistance are different measurements, so the opposite signal directions are consistent with the electrical quantity measured.

The electrical response combines bulk-fluid and interfacial processes. Electrode area, line spacing, thickness, roughness, surface condition, and excitation frequency determine the sampled volume and the relative contributions of solution resistance, double-layer capacitance, and deposit-associated impedance. Increased roughness or effective area may improve initial sensitivity but also creates more sites for conditioning-film and mineral accumulation. Passivation defines the active area and protects conductors, yet cracking, edge lift, or delamination during catheter bending can change the electrical geometry without a biological event. Biological and mineral deposits can both contribute as the interface develops. Equivalent-circuit fitting separates the response into modeled electrical components but cannot identify the material responsible. An impedance shift establishes local electrical change. Microbial composition and viability still require independent evidence [69,70,71,72,73].

Attribution to a specific biological component may require an additional recognition element. Adjacent studies have used cellulose-selective optotracing and frequency-resolved impedance fingerprinting to detect matrix-associated features or multifeature electrical patterns [73,74]. Neither approach has been evaluated for sustained operation on an integrated urinary catheter surface. Selectivity depends on target expression and probe access. Target-specific probes detect only biofilms that express the chosen marker, and conditioning films or deposits may restrict transport to the recognition layer. Long-term identification will therefore require persistent target expression and a recognition layer that remains accessible and functional during urine exposure.

Technical reliability and biological attribution require different controls. A matched reference element should share the active sensor’s urine exposure and deformation history so that wetting, bubbles, and bending can be recognized as device artifacts. Separate compositional and viability measurements are needed to attribute a remaining signal to viable biofilm. Culture alone is insufficient because a negative result cannot exclude viable-but-nonculturable cells in urinary catheter biofilms [79]. Evidence so far comes from short-term monitoring in controlled biofilm models. Specificity in the presence of conditioning films or mineral- and host-derived deposits has not been established, and long-dwell stability is unknown. A claim of CAUTI diagnosis or prediction requires catheter-episode studies with independent assessment of surface state, biofilm viability, and clinical infection. Even a valid interface signal does not indicate whether the host is inflamed. Host-biomarker sensing addresses this separate question.

### 3.4. Host-Response and Soluble-Biomarker Sensing

Host-response sensors measure soluble markers of tissue injury or inflammation, while attribution to infection requires additional evidence (Figure 2D). Direct evidence for CAUTI-oriented host-response sensing is sparse. Parolo et al. functionalized a gold microelectrode with a thiolated, methylene-blue-labeled aptamer and integrated the reagentless electrochemical sensor into a urinary catheter [75]. It reversibly monitored neutrophil gelatinase-associated lipocalin, selected as a marker of acute kidney injury, in recirculating artificial urine for 3 h. The reported architecture did not use a separate protective membrane, so probe density, exposed area, surface passivation, and recognition-layer stability directly influenced the signal. This was a short-duration demonstration of continuous in-catheter protein measurement under controlled conditions. CAUTI-associated inflammation remains to be assessed. NCT04315129 enrolled 100 participants and compared time to a Smart Catheter signal with initiation of antimicrobial treatment for suspected UTI [43]. No diagnostic-performance results have been posted, and the registry does not document independent CAUTI adjudication. It therefore records intended clinical use and study completion but provides no evidence of diagnostic validity.

Transport, dilution, and residence time can make biomarker concentration at the electrode differ from concentration at the site of release. Probe accessibility, passivation, and urine-matrix effects can also change signal gain and baseline stability. Establishing stable calibration during a long catheter dwell requires evidence beyond short-term reversibility in artificial urine. Reference elements should share the urine exposure and flow history of the active sensor. Comparison with an independent biomarker assay is also needed to separate target-dependent change from recognition-layer drift and matrix interference.

Even an analytically accurate measurement can remain clinically ambiguous because catheter-related injury can produce inflammation [8,9,10,11], while bacteriuria may occur without symptomatic infection [5,14,15]. A single inflammatory marker cannot determine the cause of injury or distinguish infection from sterile catheter trauma. Within the included studies, the dual-analyte bag platform measured pH and XOD under controlled conditions [64], whereas the in-catheter host-response platform measured a single NGAL target selected for acute kidney injury [75]. Neither was evaluated as a CAUTI-oriented panel against independently adjudicated symptomatic CAUTI or compared with a valid single-marker baseline. Thus, the incremental diagnostic value of a CAUTI panel remains untested rather than disproven. Integrated-catheter evidence remains confined to short-duration biomarker monitoring, and long-dwell clinical performance is unvalidated.

The claim boundary differs by sensing category. Urine-phase and targeted microbial systems report selected chemical, enzymatic, genomic, or growth states in sampled urine. Interface sensors show local electrical or optical change, but viable biofilm requires independent confirmation. Host-response sensors measure injury or inflammatory markers without identifying infection as the cause. None of these measurements alone establishes symptomatic CAUTI, which still requires symptoms, relevant microbiology, and independent clinical adjudication. Table 2 summarizes material architectures, integration-sensitive factors, and decisive validation needs.

## 4. Translating Catheter Sensor Signals into Supportable Claims and Actionable Alerts

**Figure 3:**
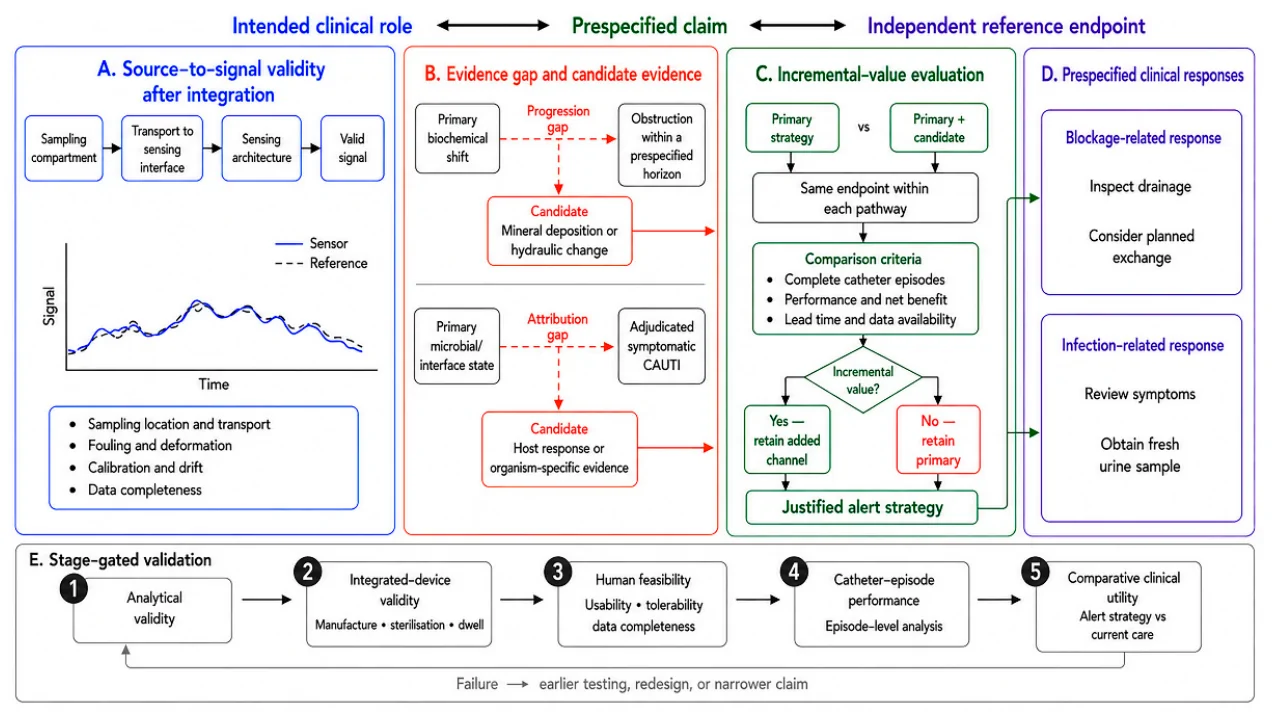
Claim-matched framework for translating catheter-sensor signals into supportable claims and prespecified clinical responses. The upper axis aligns intended clinical role, prespecified claim, and independent reference endpoint. (**A**) Source-to-signal validity after integration requires a representative sampling compartment, reliable transport to the sensing interface, an intact sensing architecture, and agreement with an independent reference while accounting for fouling, deformation, drift, and data completeness. (**B**) A progression gap separates a primary biochemical shift from obstruction within a prespecified horizon, whereas an attribution gap separates a primary microbial or interface state from adjudicated symptomatic catheter-associated urinary tract infection (CAUTI). Mineral/hydraulic measurements and host-response or organism-specific evidence are candidate channels rather than validated conclusions. (**C**) Incremental-value evaluation compares the fixed primary strategy with primary plus candidate using the same endpoint, complete catheter episodes, performance and net benefit, lead time, and data availability. (**D**) Prespecified responses are limited to drainage inspection or planned exchange for blockage-related alerts, and symptom review plus fresh urine sampling for infection-related alerts. (**E**) Stage-gated validation progresses from analytical validity to integrated-device validity, human feasibility, catheter-episode performance, and comparative clinical utility. Failure prompts earlier testing, redesign, or a narrower claim.

Clinical use depends on a clear action linked to the sensor output within the same catheter episode. For blockage, an alert could prompt inspection of the drainage system or planned catheter exchange before patency is lost. For infection-related sensing, an alert should prompt symptom review and collection of a fresh urine sample rather than antimicrobial treatment based on the sensor alone. These prespecified responses are shown in Figure 3D. Whether these actions improve care or patient outcomes has not been demonstrated.

pH-responsive blockage sensors are the only group evaluated in both continuous-flow models [47,48,49,50,51] and small human feasibility studies [41,52,53]. The 2006 and 2014 studies followed small cohorts, while URINOSTICS analyzed donated samples and observed only two blockage events [41,42,52,53]. None estimated calibrated performance across an adequately powered set of independent catheter episodes. Functional urease and responsive-release approaches remain analytical or model-based [26,27,29,44,45,46,54], and mineral or hydraulic approaches rely mainly on adjacent-device evidence [55,56,57,58,59,60,61,62]. Infection-related studies include off-device or bag-based sample assays [64,65,66,67,68], short-duration interface prototypes [69,70,71,72,73,74,75,79], and a completed registry study without posted diagnostic results against independently adjudicated CAUTI [43]. Among the included studies, robust, independently validated catheter-episode performance remains unestablished for blockage prediction or CAUTI diagnosis. Further translation depends on a claim-matched endpoint and evidence that catheter integration preserves the source-to-signal relationship. If an additional channel is used, its benefit must be shown against a fixed comparator.

### 4.1. Matching Intended Action, Claim, and Reference Endpoint

The clinical role and consequences of error determine how much evidence a sensor needs. A triage alert that prompts catheter assessment carries different risks from a result used to initiate catheter exchange or antimicrobial treatment [32,34,35]. A simple, reversible check may tolerate more false positives. Direct intervention requires stronger evidence because an incorrect result can lead to unnecessary treatment or catheter replacement. In BEST terminology, context of use links intended purpose and consequences of error to the evidence required for interpretation [32].

Claims extend from analyte detection and source-state measurement to current-state classification, future-event prediction, and clinical utility. Greater analytical sensitivity can strengthen a measurement claim but does not by itself justify a higher-level claim. Each step toward classification, prediction, or utility needs a matching reference endpoint and study design. An independent analytical method can confirm pH, urease activity, or a microbial target. Blockage, symptomatic infection, and future events require separate clinical endpoints.

In small human feasibility studies, alkaline sensor responses were not always followed by obstruction [41,52,53] (Figure 3B). This result is consistent with the pathway because urease activity and rising pH precede mineral accumulation and loss of patency. Earlier signals may allow more time for assessment. Later mineral or hydraulic changes lie closer to functional failure, although they offer less warning and remain nonspecific. The evidence supports claims of urinary alkalinization or urease activity and provides preliminary longitudinal evidence that an alkaline response may precede obstruction, but it does not establish calibrated episode-level predictive performance. A predictive claim requires a prespecified alert linked prospectively to an independently defined obstruction event within a clinically useful period [80].

The attribution branch of Figure 3B applies to infection-related sensing. The catheter-integrated NGAL aptamer sensor demonstrated reversible protein monitoring under controlled conditions but did not test whether NGAL identified symptomatic CAUTI [75]. Bacteriuria is common during prolonged catheterization [5,14,15], and catheter-related injury can produce inflammation without infection [8,9,10,11]. The initial clinical role should therefore be limited to triage or add-on use rather than standalone diagnosis. An alert could prompt symptom assessment and fresh urine sampling without directing treatment. A diagnostic claim would need clinical findings at the time of testing, relevant microbiology, and independent adjudication. Replacing the current pathway would require comparative evidence that sensor-guided use improves or at least preserves clinical decisions and patient outcomes [32,34,35].

### 4.2. Maintaining Sampling Validity and Signal Fidelity After Catheter Integration

#### 4.2.1. Location Categories, Transport, and Drainage-System Fluidics

Four sampling locations should be treated separately. Intravesical or catheter-lumen sensors encounter the freshest urine and can measure local interface change with the shortest transport delay, but they face the greatest exposure to deformation, conditioning films, biofilm, minerals, and biocompatibility constraints. Catheter-outlet, junction, or tubing sensors remain relatively accessible and sample newer urine, although wetting can be intermittent and orientation-dependent. Bag-based sensors are accessible and can support reusable readers. Bag-inlet sensors sample incoming urine after downstream transport, whereas sensors immersed in the collection bag measure pooled urine affected by mixing and storage. Off-device samples allow controlled preparation and confirmatory assays but are intermittent rather than continuous. Table 1 and Table 2 now identify location alongside material architecture so that evidence from these compartments is not treated as equivalent.

Within the catheter and tubing, sensor response reflects both convection and diffusion. At low or intermittent flow, diffusion through stagnant boundary layers and partition into a coating can dominate response time. Faster flow can thin the boundary layer but shorten contact time. Catheter eyelets, connectors, curves, and dependent tube loops can generate local recirculation or low-velocity regions, so a point sensor may sample older urine than the bulk flow suggests [81]. Bag pooling further broadens the residence-time distribution and permits continuing chemical change or microbial growth after urine leaves the bladder [12]. Validation should therefore specify flow history and residence-time distribution rather than only nominal flow rate.

Air bubbles can cover part of an electrode, alter capacitance, scatter light, interrupt wetting, damp pressure transmission, and create air locks. Drainage-bag venting changes bubble behavior and the pressure required to sustain flow [82]. During partial occlusion, local velocity through the remaining open area may rise even while total drainage becomes intermittent or falls. Upstream pressure also depends on bag height, tubing loops, compliance, catheter position, and bladder contractions. A single flow or pressure threshold may therefore misclassify drainage-system mechanics as biological progression. Test matrices should vary urine production, bag height, tubing loops or kinks, bubbles, catheter position, and graded occlusion while recording a location-matched reference measurement.

#### 4.2.2. Material Aging, Fouling, and Drift Control

Surface condition changes throughout catheter dwell. Host proteins and urinary solutes first form a conditioning film, followed by microbial attachment, extracellular matrix, mineral deposition, and mature biofilm [8,9,18,19,20,24,25]. These layers change roughness, effective electrode area, surface charge, dielectric properties, optical path length, and mass transport. For an interface sensor, deposition may be the intended measurand. For a pH, metabolite, or affinity sensor, the same deposition is a source of drift. Each design should state which surface change is signal and which is interference.

Potential antifouling strategies include hydrophilic or zwitterionic surface layers, poly(ethylene glycol)-like brushes, hydrogel or size-exclusion membranes, smooth passivation, and replaceable downstream sensing elements [28,30,31,83]. These approaches are not neutral additions. A protective layer can introduce diffusion lag, alter ion partitioning or apparent pH response, restrict access of a protein target, and crack or delaminate when a silicone catheter bends. An antifouling coating must therefore be evaluated as part of the complete transducer under representative urine, deformation, sterilization, and dwell conditions rather than inferred from protein adsorption alone.

Drift control requires more than a stable raw output. A co-located reference should share the active sensor’s substrate, electrode geometry, passivation, urine exposure, and deformation history while omitting only the recognition chemistry. Independent pH, biomarker, composition, imaging, or viability measurements are still needed to identify target-dependent change. Long-dwell reports should quantify baseline drift, sensitivity retention, hysteresis, noise, and usable-data coverage after soaking, bending, manufacture, and sterilization. A stable signal can otherwise reflect restricted transport or loss of sensitivity rather than stable analyte concentration. If a compartment is mismatched to the intended source, the claim remains limited to that compartment. If individual readings lose validity, they should be classified as invalid rather than negative.

### 4.3. Multimodal Design and Incremental Value

A multimodal design adds information only when an additional channel answers a question that the validated primary measurement cannot resolve. Figure 3C formalizes this test by comparing the fixed primary strategy with the primary strategy plus a candidate channel at the same endpoint. Apparent agreement between channels does not demonstrate incremental information, because the channels may track the same biological process or share susceptibility to flow, wetting, and fouling. Added channels can serve three distinct functions. A progression channel adds a later structural or functional measurement to determine whether an upstream change is advancing toward failure. An attribution channel adds biologically distinct evidence to identify the source of a nonspecific signal. A validity channel uses a reference or flow/contact measurement to determine whether the primary result remains interpretable.

Because channels differ in transport and response delays and may be sampled asynchronously or intermittently, multichannel analysis must align them on a common time base before agreement or incremental value is interpreted.

pH studies illustrate why a progression channel could help with blockage warning. An alkaline alert can precede obstruction, but some alerted catheter episodes do not progress to blockage [41,52,53]. A later mineral or early hydraulic measurement might separate biochemical change from progression toward loss of patency. The included studies did not test this incremental value. Human evaluations used pH-responsive strategies alone [41,52,53], while combined model-based platforms coupled the pH trigger to reporter or treatment release rather than comparing a fixed upstream sensor with and without a prespecified physical channel [27,29]. A biochemical–physical combination is mechanistically plausible, but it remains unvalidated across complete catheter episodes. Hydraulic changes also arise from low urine production, catheter position, bubbles, or other nonmineral causes, so independent evidence would still be needed to attribute progression to encrustation.

Microbial detection alone does not establish symptomatic infection. Urine-phase and organism-targeted methods report selected chemical, enzymatic, genomic, or growth states in sampled urine [64,65,66,67,68]. A host-response channel might add evidence of inflammation, but catheter-integrated data are limited to short-duration NGAL monitoring unrelated to CAUTI [75]. Organism-specific testing is available off-device [67], whereas interface sensors provide nonspecific local signals [69,70,71,72,73,74]. No study has shown that combining these measurements improves attribution, and clinical assessment with relevant microbiology remains necessary. Off-device urine tests may resolve the same uncertainty without another sensing surface, although they introduce sampling delay and workflow burden [76,77,78].

Any combined strategy should be tested against the fixed primary strategy using the same endpoint across complete catheter episodes. Predictive performance should be assessed by discrimination and, when risk is estimated, calibration. Operational evaluation should report actionable lead time, usable-data coverage, and performance under adverse flow or wetting conditions. Clinical utility requires more than an increase in area under the receiver operating characteristic curve (AUC) [35]. Decision-curve analysis can assess net benefit at relevant thresholds [84], and STARD supports transparent reporting of diagnostic-accuracy studies [33]. Results returned after the intervention window have little clinical value. A blockage result must arrive before that window closes, and an infection-related result must precede assessment and sampling decisions. Off-device confirmation remains a valid comparator when it resolves the same uncertainty with less device complexity. The preferred architecture is the least complex one that improves clinically useful performance without weakening the primary measurement.

### 4.4. An Adapted Validation Pathway from Analytical Performance to Clinical Utility

The IDEAL framework for devices (IDEAL-D) divides medical-device evaluation into idea, development, exploration, assessment, and long-term study [36]. The five-stage pathway in Figure 3E is explicitly an application and catheter-specific synthesis of IDEAL-D rather than a new general validation framework. It combines IDEAL-D staging with BEST context of use, claim-matched reference endpoints, comparative accuracy and utility, and the source-to-signal effects of catheter location and integration [32,33,34,35]. The stages move from analytical validation through integrated-device testing and human feasibility to catheter-episode evaluation and comparative clinical utility. The pathway is not strictly linear. Later testing may reveal loss of calibration, impaired catheter function, poor data coverage, or workflow failure and return development to an earlier stage.

pH-responsive blockage sensors have moved from continuous-flow models to small human feasibility studies, which show that downstream pH monitoring is feasible and that an alkaline response can precede obstruction [41,47,48,49,50,51,52,53]. These studies provide preliminary longitudinal warning evidence but do not establish calibrated episode-level predictive performance. A predictive claim now requires prospective linkage of a fixed alert rule to independently defined blockage outcomes across complete catheter episodes.

No single sample size is adequate for every intended claim. For preliminary estimation in a first fixed-threshold blockage evaluation, one illustrative precision scenario would include 50 blockage events and 50 nonblocking episodes. This is not a universal minimum. The total number of evaluable episodes should be planned from the expected event rate and may substantially exceed 100. Under a simple binomial approximation, if sensitivity and specificity are near 0.80, 50 events and 50 nonevents give approximate 95% confidence-interval half-widths of 0.11. This precision is insufficient for definitive clinical validation. A lower CAUTI event rate would require a substantially larger cohort. Confirmatory binary or time-to-event studies should use formal calculations based on the desired precision of calibration, discrimination, and net benefit, while allowing for censoring, repeated episodes within a participant, missing data, and independent external validation [85,86].

Clinical performance can be assessed only after the finished device retains its sensing behavior through manufacture and sterilization and over the intended catheter dwell. Biological safety is addressed by ISO 10993-1 and related FDA guidance [37,38], catheter requirements by ISO 20696 [63], and packaging and risk management by separate standards [39,40]. These standards govern device requirements and test methods but do not establish clinical predictive validity. Calibration and safe catheter function therefore need evidence beyond mechanical integrity and analytical accuracy. Human feasibility studies may also reveal long periods without usable data even when the device performs under controlled conditions. For continuous monitoring, episode-level coverage and the timing of invalid periods are more informative than the proportion of successful individual readings.

A catheter-episode study links a fixed sensor strategy to an independent clinical endpoint. Repeated readings from one catheter form a longitudinal record rather than independent clinical cases. Predictive performance depends on both event capture and the proportion of alerts followed by an event. The prediction horizon, repeat-alert rules, and handling of catheter replacement must be defined before analysis [80]. Reporting only the earliest successful warning interval can obscure missed events and alerts not followed by blockage. Probabilistic models require calibration and evaluation outside the development data as well as discrimination [80,87]. The Smart Catheter study illustrates a comparable endpoint limitation for infection-related sensing. It enrolled 100 participants and compared sensor timing with initiation of antimicrobial treatment, but no diagnostic-performance results against independently adjudicated CAUTI have been reported [43]. Diagnostic validity requires an endpoint that establishes whether symptomatic CAUTI was present, not enrollment alone.

Clinical utility concerns the complete alert-and-response strategy, not the sensor in isolation. Comparative studies must include invalid readings, repeated alerts, and the intended response when testing whether a fixed strategy improves care over current practice [34,35]. The comparison must capture any benefit from earlier assessment as well as unnecessary treatment or service burden caused by false alerts. Poor adherence can also make sensor failure difficult to separate from failure of the response pathway. Non-sensor catheter-management trials provide only analogous evidence, but they show that plausible interventions do not necessarily improve outcomes [88,89]. If a stage fails, the claim should narrow and the device may need redesign or discontinuation. Analytical responsiveness alone does not justify a broader clinical claim.

### 4.5. Human Factors and Implementation Barriers

A supportable claim does not by itself make a sensor usable in care. The alert pathway must specify who receives the result, how quickly it must be reviewed, which confirmatory step follows, and who is responsible for action. Repeated alerts, invalid readings, false positives, reader maintenance, and alarm fatigue can erase the value of earlier sensing. Infection-control procedures must also accommodate any reusable reader or connection without increasing manipulation of the closed drainage system.

Evaluation of human factors should include long-term catheter users, caregivers, community nurses, and hospital staff. Comfort, dignity, bag handling, visibility of alerts, digital literacy, and the burden of replacing or charging components may determine adherence. Interoperability, data governance, cybersecurity, training, procurement, reimbursement, disposal, and cost-effectiveness are further barriers for connected systems. These questions should be tested alongside technical development. A comparative clinical-utility study should evaluate the complete alert-and-response pathway against current care and measure patient outcomes, antimicrobial or catheter changes, service burden, and net benefit [34,35,36,40,84,88,89]. These implementation domains should be assessed using established medical-device usability-engineering and connected-device cybersecurity guidance [90,91].

## 5. Conclusions

The main limitation of current urinary catheter sensors is the uncertain meaning of a signal after catheter integration and its relation to the intended clinical state. pH-responsive platforms have the strongest human feasibility evidence, yet they detect urease-associated alkalinization rather than calibrated blockage risk. Infection-related measurements have a less direct relationship to a single clinical endpoint because microbial presence, catheter-surface change, and host inflammation are not equivalent to symptomatic CAUTI. Device development must preserve signal fidelity across the full catheter system and evaluate the resulting claim at its intended level of use.

The next step is prospective evaluation across complete catheter episodes. These studies should prespecify the measurement and alert strategy, use an independent endpoint matched to the claim, and reproduce representative flow and dwell conditions. Calibration and usable-data coverage should be assessed over the same period. A second channel is warranted only if it resolves a defined residual uncertainty and improves clinically useful performance over a valid single-channel or staged-confirmation comparator. The simplest architecture that provides this gain should be preferred. Episode-level evaluation should follow evidence that manufacture, sterilization, storage, intended dwell, and mechanical deformation do not compromise performance. The intended users, alert recipient, confirmatory step, and response pathway should be defined before clinical utility is tested.

## Figures and Tables

**Table 1:** Materials, sensing architectures, and supportable claims along the crystalline encrustation-to-blockage pathway.

Strategy and Pathway Position	Target Measurand	Material/Sensing Architecture, Transduction and Location	Evidence Context and Evaluated Endpoint	Current Claim Boundary	Refs.
Functional urease Upstream biochemical	Urease activity	Eu(III)-cyclen/hydrogel and enzyme-responsive polymeric reporters; luminescence or fluorescence	Analytical and controlled in vitro studies. One assay detected urease-positive bacteria within 5 h; no catheter blockage endpoint was evaluated.	Functional urease activity. Warning time and episode-level prediction remain untested.	[44,45,46]
Downstream pH Urinary alkalinization	Urinary pH	Immobilized indicator matrices, fluorescent release systems, or printed camera-read labels at the junction, tubing, or drainage bag	Continuous-flow catheter models and small human feasibility studies. Model threshold-to-blockage intervals ranged from several hours to ~43 h; human datasets contained few blockage events.	Alkalinization alert. Not calibrated blockage risk or reliable time to event.	Models [47,48,49,50,51] Human [41,52,53] Registry [42]
Catheter-proximal pH and triggered release	Local urinary pH	PVA/Eudragit S100 and multilayer pH-responsive catheter coatings or reservoirs; reporter or therapeutic release	Controlled catheter models. A signal-only coating alerted up to 12 h before blockage. Therapeutic systems prolonged patency; a phage reservoir extended time to blockage from 13 to 26 h without an independent warning readout.	Local alkalinization alert or responsive actuation. Patency extension is not warning performance, and human benefit is unknown.	[26,27,29,54]
Mineral deposition Downstream structural	Surface-associated mass loading	Polymer-coated QCM interface; resonant-frequency shift in a ureteral-stent model	Adjacent-device in vitro evidence. Dynamic surface loading was tracked during urease-induced calcium- and magnesium-phosphate precipitation; no Foley-catheter or drainage endpoint was evaluated.	Surface accumulation. Mineral attribution requires compositional confirmation and representative catheter surfaces.	[55]
Hydraulic and functional Near drainage failure	Urine volume, flow, pressure, or drainage state	Optical, pressure, or flow sensing in the catheter, tubing, bag, or adjacent monitoring systems	Continuous human monitoring has been reported in other indications, including urine output, pressure, and bladder irrigation; no study evaluated these signals against a crystalline-encrustation blockage endpoint.	Functional impairment. Not specific to crystalline encrustation or validated as blockage prediction.	Studies [56,57,58,59,60,61,62] Standard [63]

Abbreviations: PVA, poly(vinyl alcohol); QCM, quartz crystal microbalance.

**Table 2:** Substrate, functional-layer, and location determinants of signal fidelity in infection-related catheter sensing.

Category and Location	Substrate/Functional Layer/Protection	Measurand/Readout	Material and Location Limitations	Decisive Control/Validation	Refs.
Urine phase; bag or exposed catheter segment	Laser-patterned Al IDE/coil on flexible PET with stimuli-responsive polymer coatings; silicone impregnated with SNAP, with the matrix acting as reaction phase	pH or XOD → LC resonance; indole → indoxyl-red color	Easy external access; pooled or aged urine for bag-based configurations; polymer swelling and Al/coating adhesion; silicone partitioning, reagent depletion, turbidity, and reader geometry	Location-matched chemistry under representative flow and dwell; coating retention after deformation; prespecified dual-channel rule versus each single channel	[32,64,65]
Microbial target or growth; bag or off-device cartridge	MUG reagent with external FPCB optical reader; PCR products on paper discs; amine-functionalized Si/SiO_2_ microwells in a PMMA microfluidic cartridge	β-glucuronidase/growth → fluorescence; DNA → color; growth → optical interference	Post-collection growth, reagent retention, PCR inhibition, surface capture bias, optical fouling, and delay from the bladder event	Matrix controls; independent culture or molecular confirmation; prespecified thresholds and independent validation at the intended location	[66,67,68]
Interface state; catheter lumen	Cr/Au IDEs on flexible polyimide; parylene-C passivation outside the exposed sensing area; gold electrodes or recognition probes in adjacent models	Interfacial electrical properties → impedance or charge-transfer resistance; matrix features → optical/electrical pattern	Fresh local interface but overlapping protein, mineral, and biofilm effects; geometry, roughness, exposed area, bending, delamination, bubbles, and intermittent wetting	Co-located reference sharing substrate, urine, and deformation; independent composition, imaging, and viability; long-dwell testing after sterilization	[69,70,71,72,73,74]
Host response; catheter lumen	Gold microelectrode with thiolated, methylene-blue-labeled NGAL aptamer; no separate protective membrane reported	NGAL binding → square-wave voltammetric signal	Rapid local sampling but probe density and access, passivation, biofouling, recognition-layer drift, dilution, and flow dependence	Paired NGAL assay and matched reference; sensitivity retention over dwell; independent CAUTI adjudication for infection claims	[43,75]

Abbreviations: Al, aluminum; CAUTI, catheter-associated urinary tract infection; FPCB, flexible printed circuit board; IDE, interdigitated electrode; LC, inductor-capacitor; MUG, 4-methylumbelliferyl-β-D-glucuronide; NGAL, neutrophil gelatinase-associated lipocalin; PCR, polymerase chain reaction; PET, polyethylene terephthalate; PMMA, poly(methyl methacrylate); SNAP, S-nitroso-N-acetylpenicillamine; XOD, xanthine oxidase.

## Data Availability

No new data were created or analyzed in this study. Data sharing is not applicable to this article.
